# Supplementary material for: Are carbon emissions trading and green financial instruments synergistic? -Comprehensive quantitative research based on content analysis
Source: PLoS One. 2024 Mar 7;19(3):e0298601. doi: 10.1371/journal.pone.0298601 (PMC10919601; doi:10.1371/journal.pone.0298601)
Supplement: S1 Data — (ZIP) [file pone.0298601.s001.zip › Data/地级市Z_F工作报告.zip!/║■─╧/┬a╡╫╩╨/└┤╫╘╣1⁄2╓┌║┼í╛┬φ┐╦╩2╛▌═°í┐_2013.docx]

各位代表：

现在，我代表市人民政府，向大会报告工作，请予审查，并请各位政协委员和其他列席人员提出意见。

一、过去四年工作回顾

2009年至2012年，是极不平凡的四年。面对严峻复杂的宏观形势和转型发展的艰巨任务，全市上下坚持以邓小平理论、“三个代表”重要思想、科学发展观为指导，在省委、省政府和市委的坚强领导下，在市人大、市政协的监督支持下，坚定实施“科学发展，加速赶超”战略，强力推进“四化两型”建设，实现了经济社会又好又快发展，迈出了建设幸福娄底的坚实步伐。

（一）综合实力持续增强。2012年，全市完成生产总值1026亿元（预计数，下同），是2008年的1.94倍，年均增长13.2%；完成财政总收入87.7亿元、公共财政预算收入50.06亿元，分别是2008年的2.19倍、2.24倍，年均分别增长21.6%、22.4%；四年完成固定资产投资1723亿元，年均增长29.8%。新型工业加速发展。四年实现工业增加值1530亿元，年均增长16.3%。其中高耗能产业占比由68.6%下降到52%，高科技、高加工度工业占比由14.8%提高到25%。涟钢常规热轧生产线及配套改造、冷钢300万吨炼钢及配套、三一工业园一期、金电二期、国产实业一期、双峰海螺二期、湖南海螺一期、华新水泥、三一中兴二期、华润雪花啤酒、农友集团、高盛板业等一批重点项目建成投产，华菱·安赛乐米塔尔汽车板、金华特种车辆、大丰和电动汽车等项目加快推进。现代农业有效提升。四年实现农业增加值469亿元，年均增长4.3%。粮食连续9年增产增收。“三黑”等特色产业加快发展，湘村黑猪通过国家级新品种认定，响莲实业成为全国航空育种示范基地，庆阳牧业获评国家级农业产业化龙头企业，省级农产品加工龙头企业达到20家，农村合作组织797个。2012年完成农产品加工业销售收入95亿元，年均增长24.5%。三产活力逐步显现。四年实现第三产业增加值961亿元，年均增长11.7%。2012年实现社会消费品零售总额297.5亿元，是2008年的1.85倍，年均增长16.7%。全国农产品现代流通体系建设试点深入推进，“万村千乡市场工程”、“新网工程”、“双百市场工程”和中心城区农贸市场标准化改造全面实施，一批城市综合体、商业步行街、大型商业网点竣工运营。全面落实家电、汽摩下乡、家电以旧换新等政策，发放补贴近8亿元。成功创建中国优秀旅游城市，曾国藩故里、梅山龙宫、紫鹊界梯田获批4A景区，乡村旅游富民工程在全省率先启动。2012年旅游总收入达67亿元，是2008年的3.5倍。金融、会展、信息、中介、咨询等现代服务业蓬勃发展。

（二）发展基础更加坚实。四年完成基础建设投资433.3亿元。交通建设突飞猛进。沪昆客运专线、洛湛铁路娄邵线扩能改造加快推进。娄新高速建成通车，安邵、新溆、长韶娄高速开工建设，在建高速公路里程达280公里。完成干线公路改造342公里，硬化改造农村公路3829.5公里，全市公路里程达到14551公里，比2008年新增3514公里。乡镇通水泥路、建制村通公路、乡镇通客运班车均实现100%。城镇建设全面加强。城市总规修编、水府示范片区规划、万宝新城控制性详规、孙水河以南区域城市设计、北部新区城乡统筹一体化总规全面完成。全市城镇化率提高到39%，中心城区建成区面积扩大到45平方公里。国家园林城市成功创建，珠山公园、孙水公园、涟水河风光带等一批项目建成使用。吉星路、潭邵连接线、S209城区段等城市干道和出城道路建成通车，湘潭至娄底输油管道工程、湘娄邵供气管道工程、城市防洪工程等项目全面推进。国家卫生城市、全国文明城市创建进展顺利。县（市）城区不断拓展，中心镇、小城镇建设加快推进，功能不断增强。双峰县、新化县、涟源市、冷水江市先后创建省级卫生城市。新农村建设取得突破。全市78个新农村示范村建设扎实推进，冷水江市在全省率先实施统筹城乡发展整体推进城乡一体化试点。农业农村基础不断夯实，完成166座水库除险加固，治理河流126公里，改善灌溉面积16.76万亩，解决80万人的饮水安全问题，新建沼气池2万口，完成特困移民避险搬迁6590人；造林60.38万亩，森林覆盖率达到48.15%；实施土地综合整治17.7万亩，年均补充耕地1.2万亩，连续12年实现耕地占补动态平衡。园区建设成效明显。娄底经济开发区获国务院批准成功晋级国家级经济技术开发区，万宝新区、东部新区挂牌运行，娄星产业园获批省级工业集中区，双峰经济开发区获批省台资工业园。全市工业园区规划面积达到130平方公里，建成区面积达到30平方公里。园区规模工业增加值年均增长17.1%，在全市规模工业中的占比达到30.8%。

（三）社会事业协调发展。基础教育不断加强，全面免除义务教育阶段学费。四年投入6.17亿元，消除D级危房40.6万平方米，新建校舍52.5万平方米，建设合格学校390所。职成教育、高等教育、民办教育、特殊教育和学前教育协调发展。娄底职院获评国家级骨干职业学院。科技事业加快发展，荣获国家级科技奖励1项、省级科技奖励13项，专利授权3912项。医药卫生体制改革深入推进，全面实现基本药物制度全覆盖、基层医疗机构基本药物零差率销售。全民健康素质不断提升，婴儿和孕产妇死亡率低于全省平均水平。四年投入5.53亿元，新建、改扩建乡镇卫生院和社区卫生服务中心74所。文化事业再上新台阶，一批文艺作品拿回大奖，新化山歌、梅山傩戏列入全国非遗保护名录。四年新增省级文保单位20处，建成乡镇文化站86个，农家书屋3424家，兴建村级活动场所2150个。广电传媒、新闻出版、书画艺术、文学创作蓬勃发展，影响力大幅提升。省第十二届运动会筹办工作进展顺利，场馆建设加紧推进，市体育中心建成使用。全民健身活动广泛开展。人口计生工作不断创新，人口自然增长率稳定在7‰以内。

（四）民生改善成效显著。2012年城镇居民人均可支配收入19511元，农民人均纯收入4662元，分别比2008年增加7275元、1661元，年均分别增长12.4%、11.6%。先后启动保障性安居工程161个，新建廉租房20562套，新建、改建、配建公租房9734套，棚户区和国有林场改造安置38335户，完成农村危房改造27350套，解决了近15万人的住房困难。新建污水处理厂6个、垃圾填埋场5个、医疗废物处置中心1个。四年新增城镇就业人员12.4万名，安置下岗失业人员7万名，帮扶困难群众再就业2.6万名，组织37万余人参加职业技能培训。新型农村合作医疗、社会养老保险和城镇居民养老保险全面铺开，失业、工伤、医疗、生育保险进一步完善。四年投入低保资金12.89亿元，1187万人次受益；城乡医疗救助广泛开展，救助困难群众80万人次；新建和改扩建乡镇敬老院48所，入住2480人。四年投入扶贫资金13亿元，受益群众达300万人次。主要污染物减排任务全面完成，争取国家、省地质灾害和生态环境治理资金6亿元，锡矿山列为湘江流域重金属污染治理重点区域，水府庙水库生态环境保护工程启动实施，重点地区生态环境明显改善。安全生产专项治理成效明显，企业标准化特别是煤矿“四化”建设扎实推进，安全生产形势持续稳定。信访工作机制逐步完善，社会管理综合治理和应急维稳工作全面加强，人民群众安全感和满意度逐步提高。

（五）改革开放扎实推进。交通、水利、林业、农村、文化、医疗卫生、城市管理等各项改革全面推进。举办了三届湘博会、两届娄商大会，成功引进安赛乐米塔尔、国产实业、华润电力、五矿集团等战略投资者，四年签约项目510个、签约资金1756.6亿元。大力实施“走出去”战略，四年新增境外投资企业20家，投资总额6923万美元；完成进出口总额64.33亿美元，年均增长15.7%；内联引资562.9亿元，到位外资7.27亿美元，年均分别增长34.7%、25.14%。“娄底制造”对外形象不断提升，“五江”、“安石集团”、“味博士”、“好运来”成功申报国家驰名商标。银企、银政对接不断加强，招商银行、华融湘江银行、交通银行、上海农商银行等多家金融机构进驻娄底。2012年全市金融机构存款余额917亿元，贷款余额570亿元，分别是2008年的1.88倍、2.33倍，年均分别增长17.1%、23.5%。非公经济快速发展，2012年完成增加值461亿元，占全市生产总值的45%。

（六）政府自身建设全面加强。自觉接受人大及其常委会的法律监督、工作监督和政协的民主监督，四年办理市人大代表建议、议案868件，政协委员提案、建议案671件，办结率均达到100%，面商率和满意率大幅提升。普法工作全面推进，法治政府建设卓有成效，基本实现了制度化管人、管权、管事、管钱。全面实行刚性收费、刚性预算和刚性支出，建立健全了公共资源交易平台，土地拍卖、国有资产处置、项目招投标管理进一步规范。切实规范行政机关事业单位人员交流，在全省率先推行军转干部和城镇退役士兵安置考试考核制度。政府机构改革全面完成。重点领域和重点行业审计力度不断加大，政府公信力不断增强。集中整治“三乱”、“三强”等行为，发展环境不断优化。深入开展“创先争优”、“三联三转三提高”等活动，干部作风进一步转变。

国防动员和后备力量建设不断加强，驻娄人民解放军、武警部队和民兵预备役履行使命的能力持续提升，为经济社会发展和大局稳定作出了重要贡献。公安、司法、国资、统计、气象、质监、食品药品监管、民族宗教、人防、外事侨务、港澳台、档案、保密、慈善、残联、地震、仲裁、老龄、关心下一代、老区、红十字会、住房公积金、地方志和工会、共青团、妇女、儿童等各项工作均取得了新的进步。

特别是即将过去的2012年，我市克服了复杂多变的宏观形势带来的不利影响，经受住了经济下行压力不断增大的严峻考验，紧紧围绕建设幸福娄底目标，牢牢把握“稳中求好、好中求快”的总基调，积极实施六个“四年行动计划”，全力促推项目建设，全力促推产业发展，全力促推作风转变，经济社会发展取得了逆势上扬的重大突破，生产总值增长12.5%，突破千亿大关，部分经济指标在全省排位实现前移。一是全力以赴稳增长，帮扶企业取得新突破。加快实施“153211”工程，大力推进“工业四年倍增计划”和“工业企业帮扶行动”，各级干部深入一线帮助企业解决问题，有力助推了企业稳产增效。全年完成规模以上工业增加值420亿元，增长15%。全面启动“服务业发展四年行动计划”，着力推进第三产业提速发展，投资逾65亿元的中国供销湘中国际物流园等一批重大物流项目正式启动。全年完成第三产业增加值311亿元，增长12%，三产投资增长40.2%，超过全市固定资产投资增速3.2个百分点，为稳增长做出了重大贡献。二是竭尽全力扩投资，强抓项目取得新突破。坚持“以项目看能力、以项目评业绩、以项目论英雄”，以前所未有的力度开展“项目建设推进年”活动，由市级领导牵头分组挂点包干，全力以赴抓项目、上项目、促项目。全年施工项目1595个，增长24.5%，新开工项目1208个，增长31.9%；完成固定资产投资582亿元，增长37%。成功利用老工业基地建设、两型示范区建设、湘江流域重金属治理、衡邵干旱走廊治理、资源枯竭城市治理、武陵山片区发展扶贫攻坚、水府庙水库生态环境保护、资源利用“双百工程”等一系列重大政策机遇，成功争取国、省项目219个，补助资金达15亿元。三是千方百计解民忧，民生建设取得新突破。大力推进“化解大班额四年行动计划”，完成教育基础项目投资3.88亿元，启动建设项目47个，竣工15个，新增初中、小学学位8705个。建设义务教育合格学校148所，改扩建农村公办幼儿园18所，新增民办学校50所。倡议社会各界捐资助学，募集资金1.7亿元。立足解决出行难题，启动实施“交通建设四年行动计划”，娄湘公路改造等一批项目加快推进。娄新高速于12月16日竣工通车，在娄底交通史上具有里程碑意义，标志着娄底真正步入高速时代。“城乡环境整治及建设四年行动计划”和“绿化娄底四年行动计划”全面启动，“九大环保工程”加快推进，125处背街小巷改造全面完成，城中村和城乡结合部改造正紧锣密鼓推进，城乡环境明显改善。大力推进“菜篮子三年行动计划”，全市种植蔬菜面积达64.4万亩。扩增储备粮4.3万吨、食用油2万吨，粮油和食品供给得到有效保障。四是立足长远强保障，理顺机制取得新突破。制定出台《娄底市国有土地上房屋征收与补偿实施办法》和《娄底市集体土地上房屋拆迁补偿安置办法》，全面理顺征拆补偿安置机制，坚持“安置先行、同步推进”，加强失地农民的社会保障，有效化解了征拆矛盾。创新基础设施建设筹融资模式，先后采取BT、BOT、TOT等模式筹资15.28亿元，第二期13亿元市城投集团公司债券获批发行，为城市建设注入了新的动力。对市级行政事业单位国有资产、闲置土地和建设项目收费进行了集中清查、清理、清缴，整合盘活资源，为城市发展拓展了新的空间。坚持集约节约利用土地，全年获批建设用地1.39万亩，增长15%，为项目建设提供了坚实的用地保障。

各位代表！

回首过去的四年，我们经受住了百年不遇金融危机的巨大冲击，成功应对了复杂多变宏观环境的严重影响，一步一个脚印，一年一大台阶，谱写了娄底发展史上光辉灿烂的壮丽篇章。这四年，是娄底抢抓机遇最多、经济发展最好、城乡面貌变化最大的四年，是娄底基础不断夯实、优势不断提升、后劲不断增强的四年，也是娄底人民凝心聚力赶超、得到实惠最多的四年。成绩令人振奋，经验弥足珍贵。实践充分表明，只要我们始终坚持加快科学发展与立足实际主动作为紧密结合，努力提高驾驭复杂局面的能力，敢于担当、敢于变革、敢于创新，就能有效应对各种风险挑战，取得一个又一个胜利；只要我们始终坚持扩大对外开放与增强内生动力紧密结合，更加注重统筹协调，更加注重平台打造，更加注重转型发展，就能为加速赶超不断注入强大动力，实现又好又快发展；只要我们始终坚持发展经济与改善民生紧密结合，着力加强社会建设，切实解决好老百姓最关心、最直接、最现实的利益问题，就能把以人为本的科学发展观落到实处，赢得人民群众的信赖和支持。只要我们始终坚持优化人文环境与凝聚发展合力紧密结合，持之以恒理顺体制、优化机制、提升效能，就能充分调动各方面的积极性和创造性，在全市上下形成心齐、气顺、劲足的浓厚发展氛围。

各位代表！

四年的开拓奋进，四年的辉煌成就，进一步奠定了我市“科学发展，加速赶超”的坚实基础，开启了全面建设幸福娄底的崭新局面。这是市委正确领导，市人大、市政协大力支持的结果，是历届历任老领导老同志打下坚实基础的结果，是438万湘中儿女团结奋斗、顽强拼搏的结果。在此，我代表市人民政府，向全市广大工人、农民、知识分子、干部职工和企业家，向各民主党派、工商联、无党派人士、各人民团体和社会各界人士，向驻娄人民解放军指战员、武警官兵和政法干警，向关心、支持娄底建设发展的驻娄企业和单位，向在娄创业的境内外投资者和建设者，表示衷心的感谢！

我们也清醒地看到，前进道路上还存在不少困难和问题。主要是：经济总量不大，整体实力不强，发展不平衡、不协调、不可持续的问题仍然突出；产业结构不优，自主创新能力不足，调结构转方式的压力仍然很大；体制机制不顺，解决困难的办法不多，资金、用地、环境等要素保障仍然薄弱；居民收入不高，各类矛盾仍然突出，改善民生的任务仍然艰巨；安全稳定的压力不小，特别是生产安全、环境安全、金融安全和社会维稳的压力仍然较大。同时，政府职能转变不到位，一些部门和公务人员思想解放不够、工作作风不实、执行落实不力、办事效率不高，工作中推诿扯皮、行政不作为甚至为政不廉等现象还时有发生。我们将认真负责地正视这些问题，不回避、不推卸、不护短，下最大决心，尽最大努力加以解决。

二、今后五年目标和任务

未来五年，是我市“科学发展，加速赶超”、建设幸福娄底的关键时期。党的十八大提出了全面建成小康社会新的伟大目标，既为我们指明了前进方向，也提出了更高的工作要求。我们必须审时度势，抢抓机遇，奋发图强，在新的起点上努力实现新一轮更有效益、更高质量的发展。

今后五年，政府工作的总体要求是：全面贯彻落实党的十八大精神，以科学发展观为指导，紧紧围绕“三基地一中心一枢纽”的战略定位，坚持“科学发展，加速赶超”，大力推进“四化两型”建设，全面实施六个“四年行动计划”，着力稳增长、调结构、强基础、惠民生，为全面建成小康社会、努力建设幸福娄底奠定坚实基础。

经济社会发展的主要预期目标是：到2017年，生产总值达到2000亿元以上，年均增长11%以上，人均生产总值达到50000元以上；财政总收入达到165亿元，年均增长13.5%以上，其中公共财政预算收入达到95亿元，年均增长13.5%以上；固定资产投资年均增长20%以上；社会消费品零售总额年均增长15%以上；城乡居民收入年均分别增长13%和15%以上；人口自然增长率控制在7‰以内。

实现新的发展目标，要突出抓好六个方面的工作：

（一）坚持两型引领，加强生态保护，着力建设美丽娄底。以两型示范区建设为龙头，大力发展两型产业，着力推进商贸物流、金融地产、文化旅游、总部经济等产业集群发展，重点将水府示范片区建设成为先进制造业配套服务聚集区、新材料生产研发区、文化体育生态休闲旅游区和新型城镇化建设示范区。全面推进“九大环保工程”，坚持集约节约利用土地，实施最严格的水资源保护制度，坚决淘汰关闭落后产能，大力发展循环经济和节能环保产业。大力推进“城乡环境整治及建设四年行动计划”和“绿化娄底四年行动计划”，确保全市森林覆盖率达到50%以上，切实抓好锡矿山、涟邵矿区等采矿采煤沉陷区的生态修复，着力推进资水、孙水、涟水、水府庙水库以及涟钢周边环境的综合治理。让我们娄底天更蓝、地更绿、水更净。

（二）坚持基础先行，突出交通建设，着力夯实发展平台。大力推进新型城镇化建设，加快中心城区“北延南扩、东优西联、扩容提质”步伐，推进“娄—涟—冷—新”和“娄—双”城镇带建设，积极鼓励、引导和支持城镇集中连片综合开发，大力支持县（市）城区做大做强，增强辐射带动功能，以中心镇、重点镇提质扩容为重点，加强市政基础建设，着力提升城镇综合承载力和辐射力，加快农村人口市民化，确保城镇化率每年提高2个百分点以上。全面实施“交通建设四年行动计划”，加快“六纵八横”主干公路路网和洛湛铁路娄邵线、沪昆客运专线建设，以及城际快速通道、城市主干道、环线、场台站所和港口码头建设，打造“井”字型高速公路网和“米”字型铁路网，全面融入“长株潭一小时经济圈”，形成“市域一小时经济圈”。加快园区和产业集中区基础建设，抓好现有园区调规扩规和产业基础设施配套，加快打造以S312沿线为重点的现代制造业走廊和以娄双城际线两厢为重点的两型经济走廊，促进产业集中集聚发展。加强农业农村基础建设，完善城乡规划，以整体推进城乡一体化建设为方向，着力加强农村交通、水利、电力、生态环境建设，切实改善发展条件。让我们娄底发展区位更优、基础更实、后劲更足。

（三）坚持工业主导，加快产业升级，着力推进转型发展。立足资源型区域经济转型，致力推进“工业四年倍增计划”，积极发展新型工业，推进煤炭、钢铁产业转型升级和非煤矿山、建材、能源、化工、农机、煤机、电子陶瓷、特种陶瓷等传统产业改造提升，加快发展先进装备制造、新材料、新能源、电子信息、生物制药、节能环保等战略性新兴产业，扶持骨干产业、龙头企业、产业集群和产业园区做大做强，进一步壮大实体经济。大力发展现代农业，立足生猪和草食动物、优质稻、果蔬茶、南竹、中药材五大优势产业基础，着眼强龙头、扩基地，树品牌、拓市场，带农户、促增收，加快农村土地有序流转、农技推广和农村新型合作组织发展，推进农业产业化、规模化、机械化和品牌化，进一步提高比较效益，巩固农业基础地位，促进农民增收致富。加快发展现代服务业，全面实施“服务业发展四年行动计划”，继续抓好商贸流通、文化旅游、餐饮住宿、房地产等生活性服务业，大力发展现代物流、现代金融、信息科技、中介咨询等生产性服务业，努力构建与新型工业相配套、与城市发展相协调、与民生需求相适应的现代服务业体系。让我们娄底经济形态更新、产业结构更优、发展品质更高。

（四）坚持创新驱动，深化改革开放，着力激发发展活力。全面落实创新驱动发展战略，努力建设创新型娄底，加强知识产权保护，着力构建以企业为主体、市场为导向、产学研相结合的技术创新体系。在更宽领域扩大开放，加强对外交流与合作，加快战略投资者引进、央企对接、承接产业转移步伐。更加注重开展有针对性的招商活动，积极开展乡情、亲情、友情招商，支持外商来娄、娄商返乡投资兴业，努力营造亲商、安商、护商环境。更大力度解放思想，积极创新财税金融、国资管理、征地拆迁、人才培养、技术引进等领域的体制机制，努力破解资金、用地、人才、技术等方面的制约瓶颈。更深层次推进改革，继续加快项目审批、园区体制、城市管理、医疗卫生、科教文化等领域的改革，着力打造创业安心、办事顺心的良好环境。稳步推进区域发展布局优化和融城战略实施，构建以中心城区为核心，融合涟源、双峰和新化、冷水江互融的两大板块，形成“特色+板块”的发展格局。让我们娄底发展更有动力、更具活力、更富魅力。

（五）坚持以人为本，切实改善民生，着力促进社会和谐。紧紧围绕全面建成小康社会目标，用足用好政策补贴、技能培训、劳务输出、工资增长、产业富民等各项措施，努力增加城乡居民收入，让人民群众的生活更加宽裕殷实。着力落实促进就业鼓励创业的各项政策，最大限度让广大市民就近就业、自主创业，切实解决就业难题。深入推进“化解大班额四年行动计划”，着力改善农村中小学办学条件，优化教育资源配置，切实解决读书难题。深化医药卫生体制改革，进一步完善基本养老、基本医疗、工伤保险等保障制度和社会救助体系，加强保障性住房建设和棚户区、农村危房改造力度，切实解决看病和住房难题。用足用活武陵山片区区域发展与扶贫攻坚政策，全力争取项目，整合涉农资金，加大边远山区、革命老区、移民库区的扶贫开发，切实解决贫困难题。加强和创新社会管理，强化公共安全体系和企业安全生产基础建设，完善社会治安防控体系，强化司法基本保障，畅通和规范群众诉求渠道，切实维护群众权益。让我们娄底人民生活更满意、更舒心、更幸福。

（六）坚持文化为魂，提升人文素养，着力塑造文明形象。围绕创建全国文明城市，加强社会主义核心价值体系建设，全面提高市民道德素质、知识素养和人文品质。大力发展新型文化业态，加快文化与旅游、文化与体育、文化与科技深度融合，鼓励百家争鸣、百花齐放，推动文化事业大繁荣、文化产业大发展，全面增强文化软实力，扩大娄底影响力。深度挖掘本土特色文化，凝炼“真实、刚毅、执着”的娄底文化精髓，大力弘扬“坚韧、勤奋、尚德、自强”的娄底精神，全力倡导“开放包容、理性尚德、律己宽人、团结互助”的良好风尚，切实培育“娄兴我荣、娄衰我耻”的主人翁思想，在我们每个人心中树立起强烈的娄底意识，塑造文明娄底新形象。让我们作为娄底人更有归属感、更有认同感、更有自豪感。

三、2013年政府工作安排

2013年是全面贯彻落实党的十八大精神的开局之年，也是新一届政府履职的第一年。我们面临的宏观环境总体有利，但国际国内形势依然复杂多变。综合考虑，经济社会发展的主要预期目标是：全市生产总值增长12%以上，财政总收入和公共财政预算收入均增长13.5%以上，固定资产投资增长25%以上，社会消费品零售总额增长15%以上，城乡居民收入分别增长13%、18%以上，城镇登记失业率控制在4.5%以内，人口自然增长率控制在7‰以内，节能降耗完成省定目标。围绕上述目标，重点做好以下五个方面的工作：

（一）坚定不移办实事，进一步促进社会和谐。把人民对美好生活的向往作为我们的奋斗目标，扎扎实实为群众办实事、办好事、解难事。

全力破解民生难题。继续深入推进“化解大班额四年行动计划”，力争全市新增学位12400个，其中中心城区7420个，有效缓解大班额突出问题。确保吉星小学2013年春季建成开学，城南中学2014年春季正式招生。推进市儿童医院、城南医院等项目建设，加快市第一人民医院创建三级甲等医院步伐，整合市中医医院、骨伤医院创建三级甲等中医医院。加强县级公立医院、疾控中心、卫监所、乡镇卫生院、村卫生室和社区卫生服务中心建设。建立健全医疗救助制度。加大对“空巢老人”的帮扶力度，关爱留守儿童，不断提高五保补助标准。推进“菜篮子三年行动计划”，加强粮食、生猪、农资等重要生产生活资料的调控，努力稳定物价水平。全面执行新的扶贫标准，落实移民后扶政策，以边远山区、移民库区、革命老区为重点，加大政府投入、信贷投放和财政贴息规模，整合各类项目和扶贫资金，构建专项扶贫、行业扶贫和社会扶贫三位一体的大扶贫格局，力争减少贫困人口17.8万，完成特困移民避险搬迁7600人，解决2.5万移民的安全饮水问题，改善1万移民的就医条件。

全力提高保障水平。努力提高民生财政比例，不折不扣完成为民办实事项目。整合职业教育和就业培训资源，确保培训就业人员6万人以上。加强就业保障，确保全年新增城镇就业3万人以上，新增农村劳动力转移就业5万人以上。进一步扩大社会保险覆盖面，建立城乡居民大病医疗保险制度，确保生育保险和城镇医疗保险参保率达98%以上。逐步落实被征地农民社会保障。继续做好新型农村社会养老保险和城市居民养老保险工作，确保全覆盖。全力推进保障性住房建设，加大城中村、棚户区和农村危房改造力度，新建公租房9338套，棚户区改造12485套，危房改造13050户。切实加强饮用水源保护，启动娄底中心城区第二水源建设和新化县城饮用水源取水口上移，加快双峰县第二水源建设，扎实推进农村安全饮水工程和村村通自来水工程建设。

全力抓实安全监管。切实强化安全发展意识，强力推进企业标准化建设特别是煤矿“四化”建设和煤矿瓦斯治理攻坚，全面提升煤矿安全基础水平。扎实开展城市工业灾害治理。进一步增强事故救援和应急处置能力，力争全省应急救援基地落户我市。切实加强乡村公路建设、养护和城乡道路特别是娄底中心城区、县（市）城区、乡镇重点路段交通整治力度，启动实施超载治理，着力改善道路状况。加强消防安全能力建设，坚决防止重特大火灾事故发生。确保亿元GDP死亡率、十万从业人员死亡率、煤炭百万吨死亡率控制在省定目标之内。建立健全职业危害防治责任体系，全面实施粉尘危害综合治理、高毒物质和重金属重大职业危害隐患防范治理工程。加强重大疾病防控，提高突发公共卫生事件应急处置能力，有效遏制甲类传染病和大面积流行性传染病的发生。严厉打击制售假冒伪劣食品药品违法行为，加强食品药品安全各个环节的监管，确保人民群众饮食用药安全。

全力加强社会管理。坚持用群众工作统揽信访工作，完善领导干部接访制度，有效化解信访积案。加快城区电子监控系统建设，中心城区新增高清摄像头600个，基本实现电子监控全覆盖和治安防控、交通监控、城市管理“三网合一”。大力推进社区物业管理，完善政府引导、市场运作、居民自治的物业服务运行机制，完成中心城区门牌号码编排工作。加强公安基础工作，严厉打击各类违法犯罪行为，切实提高人民群众的安全感和满意度。稳定低生育水平，抓好孕前优生健康检查，着力推进青少年健康人格教育工程。抓好市文化中心建设，推进“三馆”免费开放和县乡村“两馆一站一室”建设，完成所有建制乡镇综合文化站建设，完善公共文化服务体系。加快街道办事处、居委会、村级活动场所建设，加强国有改制企业社区属地管理，加大社区队伍建设力度，进一步关心、支持基层干部，不断提高基层工作人员待遇，充分发挥他们在加强社会管理中的重要作用。

（二）坚定不移扩投资，进一步增强发展动力。把扩大有效投资作为加快发展的首要抓手，深入推进“项目建设年”活动，力争全年完成固定资产投资727亿元以上。

切实抓好项目建设。突出抓好以交通、水利为重点的基础设施项目建设。全面实施“交通建设四年行动计划”，强力推进安邵、新溆、长韶娄高速公路、沪昆客运专线、洛湛铁路娄邵线、新化资江三桥、冷水江资江二桥、大熊山连接线、S218涟源雷鸣洞至大塘、X024骡子坳至斗笠山、S312娄底涟源段、涟源冷水江段、S908新化至冷水江沿河公路、S902娄双公路和娄底中心城区内环线等重点项目建设；尽最大努力争取娄益、娄衡高速公路实质性开工建设，切实加快湄琅、龙武高速公路、洛湛铁路娄益线扩能改造等项目前期工作。继续加大水利投资，完成165座病险水库除险加固，加快推进白马大型灌区及中型灌区续建配套与节水改造，初步形成大江口水库灌区片框架，积极推进白马、双江、两丝、渠江等水源工程和大洋江航电枢纽工程建设。突出抓好以工业为重点的产业项目建设，争取涟钢2800立方米高炉技改、冷钢30万吨不锈钢复合板等项目竣工投产；全力推进华菱·安赛乐米塔尔汽车板、湖南海螺二期、创安防爆、大熊山风力发电等项目建设；着力打造汽车零部件及工程机械零部件产业园、三一产业园、冷水江循环经济产业园及光电产业园、双峰农机机电以及不锈钢产业园等一批百亿产业园区。

切实抓好项目引进。围绕国家产业政策，策划、包装、储备项目300个以上，力争更多的项目进入国省笼子。主动承接长三角、珠三角地区产业转移，引进一批汽车零配件加工项目、商贸物流项目和知名企业。继续推进央（省）企对接，重点围绕传统产业升级、产业链延长、附加值提升、资源循环利用引进战略投资者，特别注重战略性新兴产业的引进。着眼融城战略，加快娄涟路、娄双路及冷新路BT招商步伐，并尽早启动实施。积极倡导全民招商，努力营造人人参与招商、人人支持招商的良好氛围。充分发挥园区主体作用，大力开展产业招商和特色招商，提高招商引资的针对性和实效性。

切实抓好项目融资。坚持项目引资金，力争到位外资2.67亿美元，内联引资300亿元。强化银政、银企合作，规范金融监管，创建金融生态良好城市。加快引进商业银行、私募、创投等金融主体，实现村镇银行全覆盖，引进1至2家股份制商业银行，完成娄底农商行和双峰农商行筹建，启动涟源市、冷水江市、新化县农商行改制组建。力争建立1至2家高新创业投资基金。强化上市服务，力争实现上市公司零的突破。做实做强政府融资平台，积极运用BT、BOT、TOT等模式吸引社会资本参与基础设施和基础产业建设，确保新增项目融资12亿元以上。

（三）坚定不移调结构，进一步提升发展质量。把转方式、调结构、促转型作为主攻方向，进一步提质量、增效益、强产业。

改造提升传统产业。深入推进“工业四年倍增计划”和“工业企业帮扶行动”，切实解决企业融资难、用地难、用工难等突出问题。推进煤炭、煤机、农机、特种陶瓷等行业的整合，重点将246家煤炭企业整合为35家企业集团。启动市废物利用示范基地建设，加快推进冷水江市循环经济试点，支持涟钢、冷钢、湖南宜化、双峰海螺、国产实业、丰地化工等企业发展循环经济。逐步淘汰小水泥、铁合金等落后产能，强力关闭小冶炼、小造纸和小酒厂，坚决完成省定节能降耗任务。

积极培育战略性新兴产业。立足现有产业基础，有针对性地引进一批掌握核心技术、带动力强的大企业和战略投资者。选择一批成长性好、创新能力强的中小型科技企业进行重点培育，在人才引进、供地保障、资金投入等方面予以倾斜。重点支持三一中源新材料、红太阳新材料、创高铝业、方瑞钢管、三泰新材、文昌科技、映鸿科技、健缘医疗等高新技术企业做大做强。积极支持战略性新兴产业领域企业申报各类科技计划项目，突破一批关键技术，形成自主知识产权，重点支持锡矿山国家企业技术中心、涟钢企业技术中心、双峰农机机电技术服务平台建设。大力发展文化旅游产业，突出文化与旅游的融合，全面启动“三个一旅游工程”，加大曾国藩故里、梅山龙宫、紫鹊界梯田等4A景区提质升级，启动大熊山、湄江创建4A景区工作，加强仙女寨、水府庙、洪家山、龙山和世界锑都工矿旅游区的综合配套设施建设，确保清泉国际华美达、金和康年、冷水江汇美华天等五星级酒店尽快开业，加快仙女寨悠活、湘华雅阁等五星级酒店建设。结合办好大梅山文化旅游年、紫鹊界世界梯田大会、曾国藩学术研讨会等活动，搞活旅游促销，确保旅游总收入突破80亿元，增长20%以上。

加快发展现代服务业。全力推进“服务业发展四年行动计划”，以商贸物流业为重点，着力构建“一核二区四节点”现代流通体系，加快推进中国供销湘中国际物流园建设，启动中国物流娄底诚通物流园、西恩铁路物流园、万通国际汽贸城、娄底华天城、418城市综合体建设，深化全国再生资源回收利用体系和全国农产品现代流通体系建设试点，推进“农贸市场改造工程”、“万村千乡市场工程”、“新网工程”、社区便利店建设，加快发展移动电子商务及“网上供销社”，建设培育一批农产品批发大市场。巩固提升餐饮、住宿、零售等传统服务业，大力发展电子商务、服务外包、会展、中介等现代服务业。积极培育和引进有实力的知名房地产企业，促进房地产业由单纯的商住地产向金融地产、文化地产转型升级。大力发展现代金融业，加快中心城区现代金融区的改造和重构，着手谋划万宝新区金融商务区建设。把消费拉动作为重要的战略选项，全面落实“促消费、扩内需”政策，培育新的消费增长点，扩大消费稳增长、惠民生、促发展。

大力发展现代农业。全面落实惠农强农政策，促进传统农业产业做大做强，力争全年粮食播种面积400万亩以上，粮食总产165万吨以上，油料、蔬菜产量分别达到4万吨、140万吨，生猪、肉牛出栏分别达到527万头、16万头。推动农业机械化发展，力争农业耕种机械化水平达到48%以上。着力培育“三黑”等特色产业规模集群发展。加快农业产业化进程，积极发展城郊农业和休闲农业，抓好专业合作社的培育发展，切实扶持庆阳牧业、湘村高科、和盛菌业、天华牧业、百雄堂、响莲实业、广益农业、卓越粮油、汇元板业等农林产品加工龙头企业加快发展，力争农产品加工业销售收入达到120亿元以上。

鼓励发展劳动密集型产业。加大承接劳动密集型产业转移的力度，积极鼓励和引导劳动密集型产业向园区集中，向乡村集镇延伸，形成规模效应，提供更多就业岗位。扎实推进服装、制鞋、家具、食品等产业快速发展，重点支持吴哥世家扩建、金鹰服饰扩能、荣诚二期、兴昂国际四期等项目建设，力争年内竣工投产。

（四）坚定不移强统筹，进一步拓展发展空间。把统筹作为区域协调发展的重要途径，以统筹带城乡、拓空间、强基础，切实提升全面、协调、可持续发展能力。

加强城市建设和管理。以新型城镇化为引领，全力推进“城乡环境整治及建设四年行动计划”。严格规划约束，确保实现城市建设规划和新增建设用地范围控规全覆盖。中心城区以提质为重点，主攻配套、完善、提升，坚持做精、做特、做优。加速推进城市路网建设，着力拉通城市环线及主干道路网骨架，拉通娄星北路、长青西街、丽春路、桃圃街、碧溪北路等城市断头路；加快背街小巷、城乡结合部和城中村综合改造步伐；加快推进吉星220千伏输变电工程、天然气入娄、污水管网配套、城镇污水处理、垃圾填埋场环保拆迁、生活垃圾综合处理、饮用水源保护等项目建设。以冷水江市城东生态新城、涟源市新行政中心、双峰县艺芳新城、新化县北塔综合区改造等项目为重点，加快县城、中心镇发展步伐，构建中心城区、县城、中心镇梯度推进、优势互补、协调发展的城镇体系。以创建国家卫生城市为契机，积极推行城市管理“运营市场化、作业精细化、管理网络化、考核科学化”，全面落实“门前三包”制度和严管重罚措施，切实加强城市保洁，推行中心城区“净化、绿化、亮化、序化、美化”工程，从根本上改善城区环境面貌。

推进新农村建设步伐。继续推进冷水江市统筹城乡发展整体推进城乡一体化试点和“百城千镇万村”新农村建设工程试点，着力抓好农村道路改造、房屋风貌改造、改水改厕、植树造林、沼气开发等工作。强力推进农村环境卫生专项整治，积极推进生猪和蔬菜清洁生产。扎实推进乡村清洁工程，实现“四清三基二统一化”目标。加强农村建房规划引导和控制，规范村民适度集中建房。全面加强农村公共设施建设，建立健全农村社会化服务体系，加快城市公共设施和公共服务向农村延伸。

加大环境保护力度。以“九大环保工程”为统揽，切实抓好湘江流域重金属治理、水府庙水库和孙水、涟水、资水流域的环境治理。推进“绿化娄底四年行动计划”，加快“三边一区”和矿山、湿地植被恢复，抓好联村建绿和造林绿化，完成娄新高速绿色通道建设。抓好森林防火和林业有害生物防治，严厉打击乱占林地、乱采滥挖、乱捕滥猎等违法行为。启动涟钢周边环境综合整治，加快矿山环境治理。严格环保审批，全面完成节能减排任务，进一步加强工业烟（粉）尘、工业废水、垃圾倾倒、建筑施工扬尘和机动车尾气治理，努力改善城乡环境质量，确保人民能喝上干净的水、呼吸到新鲜的空气、吃上放心的食品。

加快园区和示范区建设。统筹协调园区和示范区产业布局，逐步理顺体制机制。充分发挥娄底经济开发区国家级品牌优势，不断壮大精品薄板及深加工、工程机械及汽车制造、电子信息等主导产业，带动县（市、区）产业园区加快发展。重点支持娄底经济开发区中小企业孵化园200万平方米标准厂房建设，确保各县（市、区）经济园区建设标准厂房20万平方米以上。加快万宝新区建设步伐，力争完成起步区4平方公里基础建设和百亩组团2平方公里产业园区建设，引进一批投资10亿元以上的项目，形成示范带动效应。重点抓好高铁南站站场及站前广场、娄星南路、仙女大道等基础设施建设，加快推进新世界建材城、豪布斯卡国际综合街区等重点项目建设。进一步创新理念，完善顶层设计，加快推进东部新区两型示范建设。

（五）坚定不移抓改革，进一步汇聚发展活力。把改革开放作为破解发展瓶颈的重要手段，以改革增活力、强动力，以开放促改革、促发展。

扎实推进各类改革。深化两型社会综合配套改革，健全与之相适应的体制机制。深化文化体制改革，进一步理顺文化管理体制和文化产品生产经营体制。深化医药卫生体制改革，全面推行村卫生室实施基本药物制度，全面启动公立医院改革，巩固新型农村合作医疗，确保农民参合率稳定在98%以上。深化人事制度改革，完善公务员考核制度，深入推进事业单位改革，创新军队转业干部、随军家属和城镇退役士兵安置方式，拓宽人才引进渠道。深化教育综合改革，逐步理顺教育管理体制，促进城乡教育均衡发展和民办教育规范协调发展。以培养实用型人才为重点，大力推进职业教育人才培养模式的改革。积极稳妥推进水利和国有林场改革。深化财税管理改革，进一步健全预算编制、执行和管理制度，稳步推进预算公开，加强支出绩效管理，提高财政资金使用效益。完善国有资产监管体系，确保国有资产保值增值。深化资源价格改革，继续稳妥推进水、电、气等资源价格和污水、垃圾处理等环保收费改革，充分发挥价格杠杆在节能减排方面的积极作用。

提升对外开放水平。加快承接产业转移，积极开展对外贸易，大力发展加工贸易和服务外包，培育新的外贸增长点。办好第四届湖南省农业机械、矿山机械、电子陶瓷产品博览会，构建区域性经贸平台。推进“品牌兴贸”、“科技兴贸”战略，加快拓展新兴市场，组织境外展销，开展电子商务，引导企业建立境外销售平台。加快娄底铁路口岸、进出口基地、服务外包基地建设，同步推进海关、进出口检验检疫等配套建设，夯实外贸基础条件。大力推进“走出去”战略，鼓励企业境外投资、承揽工程，力争新增境外投资企业5家以上。建立市级对外劳务合作服务中心，扩大劳务输出。毫不动摇鼓励、支持和引导非公经济发展，进一步增强经济发展活力。

不断优化创业环境。以政府信用、企业信用、个人信用为主体，健全社会信用体系。继续从严打击强装强卸、强买强卖、强揽工程等违法犯罪行为，切实优化项目施工环境。严厉打击逃骗抗税、制假售假、围标串标、欺行霸市等违法犯罪行为，着力优化市场环境。加快政策性文件的立、改、废工作，建立面向全社会的信息公开制度、政策制定意见征询制度和安全维稳风险评估制度，积极营造宽松的政策环境。以深化行政审批制度改革和清理规范行政事业性收费为重点，规范政府行政行为，简化办事程序，提高办事效率。对已经出台的各项优惠政策，坚决兑现，绝不含糊；对严重影响发展环境的人和事，坚决查处，绝不姑息。

与此同时，认真开展国防教育，扎实推进民兵转型发展，不断完善人防设施，巩固深化军政军民关系，积极促进军民融合式发展，努力提高国防动员和后备力量建设质量。切实做好第十二届省运会筹备工作，按进度完善基础设施建设。加强统计基础工作，抓好第三次经济普查。统筹抓好民族宗教、港澳台、外事侨务、地方志、住房公积金、档案、保密、气象、水文、仲裁、防震减灾、老龄、慈善、残联和红十字会、工会、共青团、妇女等各方面工作。

四、加强政府自身建设

把人民群众的呼声作为第一信号，把人民群众的评价作为第一标尺，出实招、重实干、求实效，切实提高政府执行力、推动力、公信力。

切实解放思想。坚决破除靠山吃山、小富即安的思想，积极培育创先争优、敢闯敢拼的意识，以跳起来摘桃子的勇气奋发进取，以只争朝夕的激情推进工作。坚决破除心胸狭隘、只顾眼前的思想，积极培育胸怀全局、着眼长远的意识，以开阔的视野研判形势，以战略的眼光谋划未来。坚决破除循规蹈矩、死守惯例的思想，积极培育开拓创新、敢为人先的意识，敢于冲破阻碍发展的惯例思维、习惯做法，敢于革除影响发展的体制机制、条条框框。只要法律和政策没有明文禁止，只要其他地区已有先例，只要有利于促进我市发展、增进人民福祉，就要敢于开拓，敢于担当，敢于突破。

严格依法办事。全面加强法治政府建设，坚持用制度管人、管权、管事、管钱，确保一切权力在阳光下运行，让政府行政行为更加公开、公平、公正。自觉接受人大及其常委会的法律监督、工作监督和政协的民主监督，认真办理人大代表议案、建议和政协委员提案、建议案，广泛听取民主党派、工商联、无党派人士和人民团体的意见，集中精力把定下来的事情抓落实，把人民群众关心、关注的事情办到位。严格落实党风廉政建设责任制，全面加强对财政资金、重大项目的审计监督和建设工程、公共资源交易、政府采购等方面的监管，严肃查处贪污腐败、以权谋私、索拿卡要等违法违纪案件，切实做到干部清正，政府清廉。

大力改进作风。弘扬求真务实、真抓实干的作风，力戒形式主义、官僚主义。进一步精简会议、文件，规范检查评比，可开可不开的会议坚决不开，可发可不发的文件坚决不发，没有市委、市政府批准，不搞单独的检查评比。提倡艰苦奋斗，厉行勤俭节约，没有市委、市政府安排，不参与奠基剪彩题词。市内活动不搞迎来送往，不搞相互吃请，不搞交通管制，不搞警车牵引。深化行政审批制度改革，提高办事效率，进一步清理、减少和调整行政审批事项，全面推行服务承诺、首问责任、限时办结制度，加强绩效管理，强化督促考核，铁腕整治“中梗阻”、“庸懒散”，严厉查处推诿塞责、相互扯皮、办事拖沓的人和事，做到政令畅通、令行禁止。坚持深入基层，密切联系群众，多蹲点调研，多下访解难，多结对帮扶，多上门服务，真正让政府服务在基层，使问题解决在基层，把威信树立在基层。

各位代表！昨天的辉煌已经载入史册，未来的伟业需要我们共同开创。站在新的发展起点，让我们在市委的正确领导下，凝聚各方力量，坚定发展信心，顽强拼搏，一往无前，奋发图强，为全面建成小康社会，加快建设幸福娄底，创造更加美好的明天而努力奋斗！
